# Cardiovascular magnetic resonance stress perfusion compared to single-photon emission computed tomography (SPECT) in patients with left main stem disease: a CE-MARC substudy

**DOI:** 10.1186/1532-429X-14-S1-O93

**Published:** 2012-02-01

**Authors:** John P Greenwood, Ananth Kidambi, Arshad Zaman, Neil Maredia, Manish Motwani, Catherine J Dickinson, Julia Brown, Jane Nixon, Colin Everett, Stephen G Ball, Sven Plein

**Affiliations:** 1 Cardiology, Multidisciplinary Cardiovascular Research Centre & Leeds Institute of Genetics, Health and Therapeutics, Leeds, UK; 2 Division of Medical Physics, University of Leeds, Leeds, UK; 3 Leeds Teaching Hospitals NHS Trust, Leeds, UK; 4 University of Leeds, Leeds, UK

## Summary

We compared detection rates for cardiovascular magnetic resonance (CMR) perfusion and single photon emission tomography (SPECT) in the subset of patients from the CE-MARC study with significant left main stem (LMS) disease. Detection rates for LMS disease by CMR perfusion were higher than for SPECT, and CMR identified a classical LMS pattern with higher frequency. Visual perfusion defects occurred with similar frequency in patients with ≥50% and ≥70% LMS stenosis.

## Background

Left main stem (LMS) disease is found in approximately 5% of patients with stable angina. Three-year survival in patients with >50% left main stenosis may be as low as 50%. Single photon emission tomography (SPECT) fails to detect ischemia in up to 15% of LMS stenoses, and identification of the ‘classical’ pattern of both left anterior descending (LAD) and circumflex (LCx) coronary territory ischaemia is lower still. To date, the utility of cardiovascular magnetic resonance (CMR) perfusion in LMS disease is poorly established. The CE-MARC study was a prospective study of 752 patients with suspected coronary artery disease, enrolled to undergo CMR, SPECT and X-ray coronary angiography. We assessed the diagnostic performance of SPECT and CMR to detect LMS disease in the group of CE-MARC patients with ≥50% LMS disease on quantitative X-ray angiography. We also compared subsets of patients with ≥50% and ≥70% LMS stenosis.

## Methods

All patients with LMS disease ≥50% on quantitative angiography were identified from the CE-MARC study. All patients had undergone adenosine stress perfusion by CMR and SPECT and also invasive X-ray angiography [1]. By visual analysis we compared detection rates for LMS disease from the CMR and SPECT perfusion studies.

## Results

**Figure 1:**
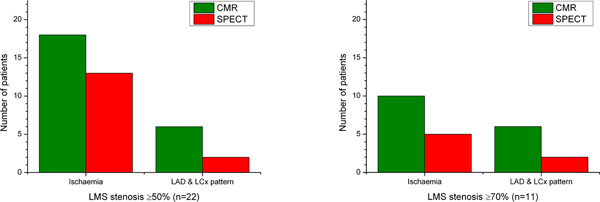
Detection rate of CMR and SPECT in LMS disease. Patients with LMS stenosis ≥50% (n=22) and the subset with stenosis ≥70% (n=11) are shown.

Of 23 patients in the CE-MARC cohort with LMS stenosis ≥50%, one patient could not be analysed. CMR identified evidence of inducible perfusion defects in 18/22 (82%) of the LMS group; SPECT identified 13/22 (59%). For CMR and SPECT respectively, inducible perfusion defects were found in both LAD and LCx territories for 6/18 (33%) and 2/13 (15%). Only one patient had normal perfusion analyses (false negative) for both CMR and SPECT. Of 11 patients with ≥70% LMS stenosis, 10 (91%) had inducible perfusion defects with CMR vs. 5 (45%) with SPECT. Six (55%) vs. 2 (18%) had a LAD and LCx disease pattern. Perfusion abnormalities were detected with similar frequency in ≥50% and ≥70% groups by both CMR (p=0.64) and SPECT (p=0.49). Figure 1 summarises the detection rate of CMR and SPECT in LMS disease.

## Conclusions

CMR stress perfusion imaging identifies ischaemia in a higher proportion of patients with significant LMS disease than SPECT, and identifies a ‘classical’ LMS pattern with higher frequency. Perfusion abnormalities are detected with similar frequency in patients with ≥50% and ≥70% LMS stenosis.

## Funding

The CE-MARC study was funded by a British Heart Foundation Programme Grant (RG/05/004). S.P is funded by British Heart Foundation fellowship (FS/10/62/28409).

## References

[B1] Greenwood JP CE-MARC: A Prospective Evaluation of Cardiovascular Magnetic Resonance and Single-Photon Emission Computed Tomography in Coronary Heart Disease The Lancet 2011 in press 10.1016/S0140-6736(11)61335-4 PMC3273722 22196944

